# Differential Alterations of the Mitochondrial Morphology and Respiratory Chain Complexes during Postnatal Development of the Mouse Lung

**DOI:** 10.1155/2017/9169146

**Published:** 2017-12-19

**Authors:** Natalia El-Merhie, Eveline Baumgart-Vogt, Adrian Pilatz, Susanne Pfreimer, Bianca Pfeiffer, Oleg Pak, Djuro Kosanovic, Michael Seimetz, Ralph Theo Schermuly, Norbert Weissmann, Srikanth Karnati

**Affiliations:** ^1^Institute for Anatomy and Cell Biology II, Division of Medical Cell Biology, Justus Liebig University, Giessen, Germany; ^2^Department of Urology, Pediatric Urology and Andrology, Justus Liebig University, Giessen, Germany; ^3^Excellence Cluster Cardio-Pulmonary System (ECCPS), German Lung Center (DZL), Universities of Giessen and Marburg Lung Center (UGMLC), Justus Liebig University, Giessen, Germany

## Abstract

Mitochondrial biogenesis and adequate energy production in various organs of mammals are necessary for postnatal adaptation to extrauterine life in an environment with high oxygen content. Even though transgenic mice are frequently used as experimental models, to date, no combined detailed molecular and morphological analysis on the mitochondrial compartment in different lung cell types has been performed during postnatal mouse lung development. In our study, we revealed a significant upregulation of most mitochondrial respiratory complexes at protein and mRNA levels in the lungs of P15 and adult animals in comparison to newborns. The majority of adult animal samples showed the strongest increase, except for succinate dehydrogenase protein (SDHD). Likewise, an increase in mRNA expression for mtDNA transcription machinery genes (*Polrmt*, *Tfam*, *Tfb1m*, and *Tfb2m*), mitochondrially encoded RNA (*mt-Rnr1* and mt*-Rnr2*), and the nuclear-encoded mitochondrial DNA polymerase (POLG) was observed. The biochemical and molecular results were corroborated by a parallel increase of mitochondrial number, size, cristae number, and complexity, exhibiting heterogeneous patterns in distinct bronchiolar and alveolar epithelial cells. Taken together, our results suggest a specific adaptation and differential maturation of the mitochondrial compartment according to the metabolic needs of individual cell types during postnatal development of the mouse lung.

## 1. Introduction

Mitochondria, commonly referred to as the “powerhouse” of the cell, are involved in energy production, *β*-oxidation of fatty acids, calcium buffering, cell signaling, proliferation and apoptosis, embryonic development, and general body ageing [1, 2]. Structurally, mitochondria differ from other cell organelles by possessing four distinct membrane compartments: the outer mitochondrial membrane (OMM), the intermembrane space (IMS), the inner mitochondrial membrane (IMM) that forms invaginations called cristae, and the matrix [3, 4]. The most important role of mitochondria is the production of nicotinamide adenine dinucleotide (NADH) and adenosine triphosphate (ATP) [5–7]. The respiratory chain, comprising five protein complexes and residing in the inner mitochondrial membrane (IMM), is responsible for the generation of ATP by oxidative phosphorylation (OXPHOS) [8]. Five respiratory chain complexes are known as complex I (NADH-coenzyme Q oxidoreductase), complex II (succinate-coenzyme Q oxidoreductase), complex III (coenzyme Q-cytochrome c oxidoreductase), complex IV (cytochrome c oxidase), and complex V (ATP synthase) [9]. Most OXPHOS complexes are encoded by both mitochondrial and nuclear subunits with the exception of complex II, which is solely encoded by nuclear genes [10]. Moreover, mitochondria contain in their matrix a circular genome (mtDNA) that is essential for their function in oxidative phosphorylation. The mtDNA encodes 13 polypeptides of complexes I, III, IV, and V, 2 ribosomal RNAs, and 22 tRNAs [11].

Mitochondrial metabolic pathways are interlinked, and the whole mitochondria interact with other organelles, such as the endoplasmic reticulum and peroxisomes, facilitating intracellular/interorganellar communication and influencing cellular metabolic functions [12, 13]. The mitochondrial membrane potential is essential for the normal functioning of the mitochondrial respiratory chain, in which even under physiological conditions reactive oxygen species (ROS) are released [14]. Under pathological conditions, higher generation of ROS (H_2_O_2_) combined with NO leads to the production of peroxynitrite (ONOO^−^) or other RNS species [15, 16]. Higher NO levels impair mitochondrial respiration and membrane potential and inhibit superoxide dismutase (SOD2), leading to a higher superoxide release [17, 18]. To combat higher generation of ROS, mitochondria employ a complex network of enzymatic and nonenzymatic defense systems. These antioxidant systems include enzymes localized within the mitochondrial matrix such as manganese-containing superoxide dismutase (MnSOD), glutathione peroxidase (GPx), glutathione reductase (GR), thioredoxin II (Trx II), and peroxiredoxins I and III (Prxs I and III) as well as the enzymes localized within the mitochondrial intermembrane space such as copper/zinc (Cu/Zn) SOD [19–23]. Mitochondria are also involved in acetyl-CoA production and in calcium metabolism [24, 25].

Many energy-demanding physiological processes are initiated at birth, leading to the modification of the metabolic pathways important for energy production. This modification, which triggers the switch from glycolysis to respiration, depends on the maturation of mitochondria [26, 27]. It is known that the acquirement of functional mitochondria after birth is an important homeostatic process allowing newborn mammals to adapt for the extrauterine life in an environment with high oxygen levels. Therefore, the induction of many mitochondrial enzymes occurs during the first hours of postnatal life [28]. Further, during postnatal lung development also, mitochondria of nonciliated bronchiolar epithelial cells and type II alveolar epithelial cells (AECII) undergo extensive remodeling by significant alterations in their morphological appearance (volume density, size, number, and distribution) [29–32].

It is becoming increasingly clear that mitochondrial dysfunction may promote or predispose to the onset of lung diseases. Indeed, mitochondrial dysfunction with reduced levels of respiratory complexes has been shown in COPD and non-COPD smokers as well as in bronchial epithelial cells in mice suffering from asthma [33–38]. Given the extensive usage of wild-type and various genetically modified mice to understand the pathophysiological changes, it is essential to investigate mitochondrial biogenesis, metabolism, and maturation during regular postnatal lung development of wild-type mice. However, to date, no detailed combined molecular and morphological analysis has been performed on the mitochondrial compartment during postnatal development of the mouse lung. Postnatal alterations of the mitochondrial compartment and its respiratory function have been mainly described in rat liver and muscle [28, 39–44], pig skeletal muscle [45], rat, rabbit, and bovine hearts [43, 46–49], mouse blood lymphocytes [50], and rat and mouse brain [51–53]. Therefore, here, we analyzed mitochondrial alterations during postnatal development (newborn, P15, and adult) of the mouse lung. Our results revealed an upregulation of most mitochondrial complexes (complexes I, III, IV, and V) at the protein and the mRNA levels in the lung tissue samples of P15 and adult in comparison to newborn mice. These results were corroborated by the upregulation of mRNAs for mtDNA transcription machinery genes, mitochondrially encoded RNA, and mitochondrial DNA polymerase from the lungs of the P15 and adult mice in comparison to the lungs of the newborn mice. Interestingly, a clear difference was observed in the SDHD protein abundance pattern that peaked in the lungs of P15 animals and then decreased in the adult animals.

## 2. Materials and Methods

### 2.1. Experimental Animals

Three groups of C57BL/6J mice with different postnatal ages were used in this study: eighteen newborn pups (P0.5) of four pregnant dames as well as 18 male mice (9 mice at postnatal day and 15 and 9 adult mice at 12 weeks of age). The mice were obtained from Charles River (Sulzfeld, Germany) and were housed after the delivery in cages at the central animal facility (Zentrales Tierlabor (ZTL)) of the Justus Liebig University (Giessen, Germany). They were maintained under standard environmental conditions with a 12-hour light/dark cycle at 23°C ± 1°C and 55% ± 1% relative humidity. Animals had access to normal laboratory diet and food ad libitum. All animal experiments in this study were approved by the German Government Commission of Animal Care (Regierungspraesidium Giessen, Germany, permit number V 54-19 C 20/15 c GI 20/23).

### 2.2. Perfusion Fixation, Sampling, and Tissue Processing for Routine Transmission Electron Microscopy (TEM)

The detailed protocol for perfusion fixation with a 1.5% paraformaldehyde (PFA)/1.5% glutaraldehyde (GA) in 0.15 M HEPES buffer (pH 7.4), sampling, and tissue processing of lungs for routine transmission electron microscopy (TEM) was described previously [54]. Fixed lung tissue blocks of 1 mm^3^ of the newborn, P15, and adult animals were embedded into the epoxid Agar 100 Resin® (Agar, Essex, England) and polymerized for 3 days at 60°C. Embedded tissue blocks were trimmed with a diamond trimmer (Reichert TM 60, Austria), and then ultrathin sections (80 nm) were cut with a Leica Ultracut E ultramicrotome (Leica, Nussloch, Germany). The cut sections were contrasted with uranyl acetate (2 min) and lead citrate (45 s) and thereafter examined with a LEO 906 transmission electron microscope (LEO Electron Microscopy, Oberkochen, Germany) equipped with a 2k-camera (TRS, Troendle systems).

### 2.3. Postembedding Immunoelectron Microscopy of Mitochondrial Proteins

The detailed protocol for perfusion fixation with 4% (*w/v*) paraformaldehyde (PFA)/0.05% (*v/v*) glutaraldehyde (GA)/PBS, processing of lungs, embedding into LR white was described previously [54–56]. Briefly, ultrathin sections of lung tissue were incubated overnight in a wet chamber with primary antibodies (Table 1) in 0.1% BSA-c (Aurion) in PBS containing 0.05% Tween 20. On the next day, the sections on grids were washed 6 times on a series of 0.1% BSA-c (Aurion) in PBS containing 0.05% Tween 20 drops and then incubated for 120 min with ultrasmall immunogold goat anti-rabbit Fab fragments (Aurion) in 0.1% BSA-c (Aurion), diluted with 1 : 400 in PBS containing 0.05% Tween 20. Then, the grids with sections were rinsed shortly (3 × 3 min) with 0.1% BSA in PBS containing 0.05% Tween 20 followed by the washing with PBS (3 × 3 min). Thereafter, the antigen-antibody complexes were fixed for 10 min with 2% glutaraldehyde in PBS. The fixative was washed away with a drop series of PBS (3 × 3 min), followed by (6 × 3 min) aqua dest. Silver intensification was done according to the method of Danscher in a light tight box for 25 min at RT [57]. Thereafter, grids with sections were washed for 6 times (3 min each) with aqua dest. Sections were contrasted with uranyl acetate (2 min) and lead citrate (45 s) and thereafter examined with a LEO 906 transmission electron microscope (LEO Electron Microscopy, Oberkochen, Germany) equipped with a 2k-camera (TRS, Troendle systems).

### 2.4. Immunofluorescence on Paraffin-Embedded Tissue

The detailed protocol for perfusion fixation, paraffin embedding, sectioning of lung tissue, and subsequent immunofluorescence for newborn, P15, and adult lungs was described previously [55]. Briefly, perfusion-fixed lungs (4% PFA in PBS, pH 7.4) were embedded into paraffin (Paraplast, Sigma-Aldrich, St. Louis, MO, USA) using an automated vacuum tissue processor (Leica TP 1020) and sections (2-3 *μ*m) were cut with Leica RM2135 rotation microtome and processed for double immunofluorescence. The dilutions of the primary and secondary antibodies used are listed in Table 1. Negative controls for secondary antibody reaction were processed in parallel by addition of PBST buffer instead of the first antibodies. Nuclei were visualized with 1 *μ*M TOTO-3 iodide (molecular probes) for 10 min at RT. Samples were analyzed by confocal laser scanning microscopy (CLSM) with a Leica TCS SP5 (Leica Mikrosysteme Vertrieb GmbH, Wetzlar, Germany). All images were processed with Adobe Photoshop CS5.

### 2.5. Preparation of Whole Lung Homogenates for Protein Analysis (Western Blotting)

For Western blotting, 3 lungs each from the newborn, P15, and adult mice were collected and stored at −80°C prior to homogenization. The lung tissue of each group was cut into small pieces and homogenized in 2 ml ice-cold homogenization buffer as previously described [55].

### 2.6. Western Blotting

For Western blot analysis, 50 *μ*g of lung homogenates from the newborn, P15, and adult mice was separated on 10% SDS-polyacrylamide gels using a Bio-Rad gel electrophoresis apparatus (Bio-Rad, München, Germany) as previously described [55]. Dilutions of primary antibodies used are listed in Table 1.

### 2.7. RNA Isolation

For RNA isolation, 100 mg of frozen lung tissue was homogenized with an IKA T 25 ULTRA TURRAX (IKA, Germany) in 1 ml RNAzol (RNAzol® RT, Sigma-Aldrich). Then, 0.4 ml of RNase-free water per ml of RNazol was added and left for 15 min at RT. The lysate was centrifuged at 12,000*g* for 15 min and the supernatant was transferred to a fresh tube to which an equal volume of 100% isopropanol was added. After that, the lysate was centrifuged at 12,000*g* for 10 min and the supernatant was discarded. The RNA pellet was washed twice with 0.5 ml of 75% ethanol per ml of supernatant. The RNA was centrifuged at 8000*g* for 3 min at RT, and the ethanol was removed. Finally, the RNA pellet was solubilized in RNase-free water at concentration of 1-2 *μ*g/ml. The purity and quantification of RNA was determined with a spectrophotometer.

### 2.8. cDNA Synthesis

1 *μ*g total RNA from adult, P15, and new born lungs was reverse transcribed to cDNA using 1 *μ*l of (dT) 12–18 primer (Invitrogen, Germany) and 1 *μ*l of SuperScript™ II Reverse Transcriptase (RT) Kit (Invitrogen, Germany) according to the manufacturer's protocol. The reaction was incubated in a Biometra Trio Thermocycler (The Netherlands). The qRT-PCR of target genes, described in Table 2, was performed in the iCycler iQ5™ Real-Time PCR Detection System (Bio-Rad, USA). The reactions were set up with the SYBR™ Green PCR mix (Life Technologies) according to the manufacturer's protocol. The PCR cycle consisted of an initial cycle of 95°C for 3 min followed by 42 repeated cycles of 95°C for 15 s, 60°C annealing temperature for 30 s, and the primer extension at 72°C for 1 min. The real-time PCR primer pairs used in this study are listed in Table 2. All reactions were run in triplicates. Calculation of the relative gene expression was done by the 2^−ddC^_T_ method, where dC_T_ = (C_T_target gene − C_T_internal control gene) using GAPDH as an endogenous control.

### 2.9. Statistics

Data are expressed as mean ± standard deviation. Differences between groups were evaluated by Student's unpaired *t*-test and one-way analysis of variance (ANOVA) using Tukey's test. Data were considered statistically significant if *p* < 0.05.

## 3. Results

### 3.1. Abundance of Mitochondrial Proteins in Lung Homogenates

**Figure 1:**
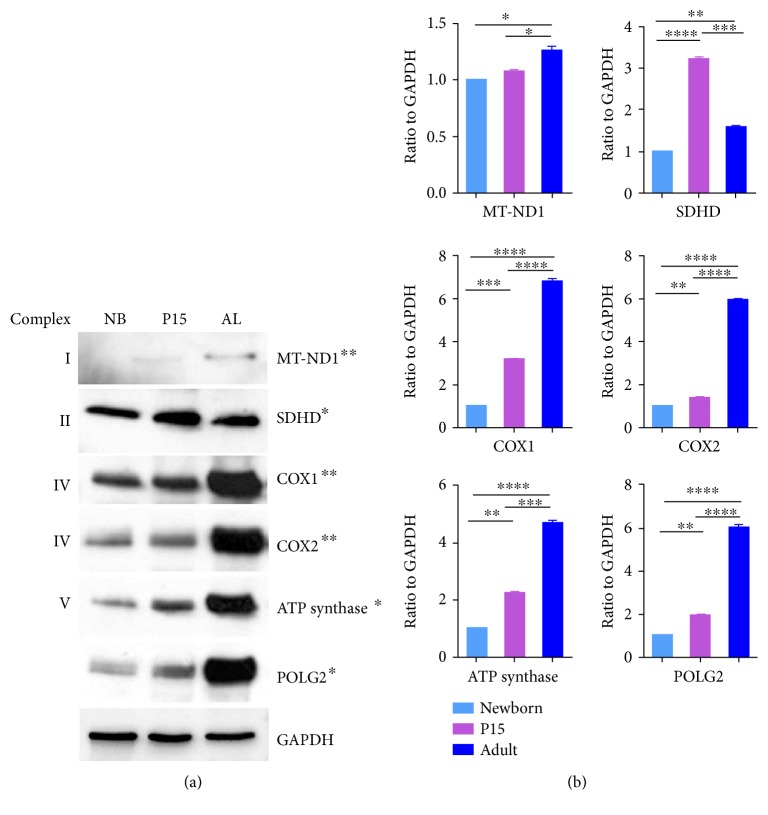
Western blot analyses of distinct mitochondrial proteins in lung homogenates from newborn, P15, and adult mice. (a) Following the homogenization, 50 *μ*g of protein isolated from the lungs of each group (NB, P15, and adult) was resolved by 10% SDS-polyacrylamide gel electrophoresis, and the blots were immunostained with anti-MT-ND1, anti-SDHD, anti-COX1 (OXPHOS complex IV subunit I), anti-COX2 (OXPHOS complex IV subunit II), anti-ATP synthase (ATP5b), and anti-POLG2. GAPDH was used as a loading control. ^∗^Nuclear-encoded protein. ^∗∗^Mitochondrially encoded protein. (b) Bar graphs summarizing normalized data. *p* values were calculated by the one-way ANOVA using Tukey's test. *n* = 3; ^∗^ *p* ≤ 0.05, ^∗∗^ *p* ≤ 0.01, ^∗∗∗^ *p* ≤ 0.001, and ^∗∗∗∗^ *p* ≤ 0.0001.

**Figure 2:**
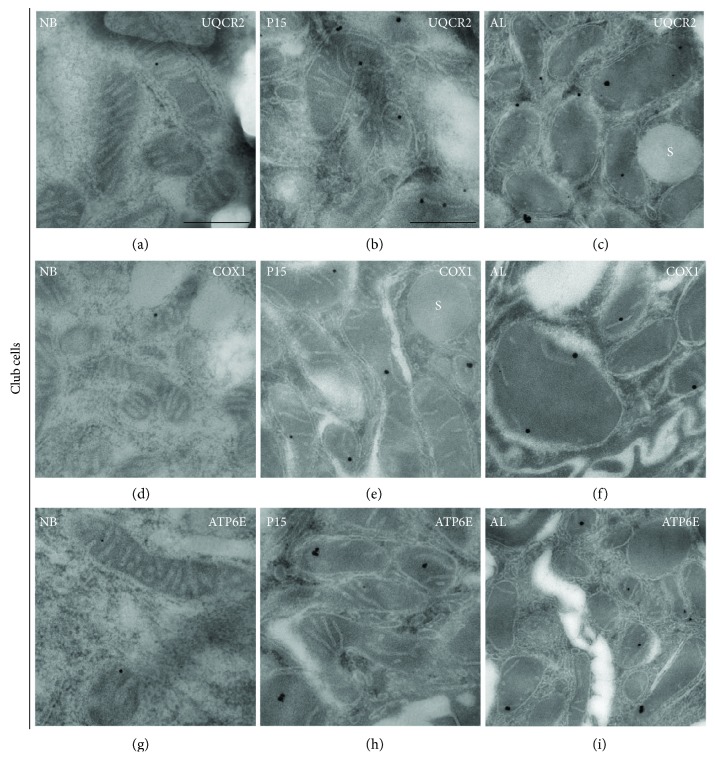
Electron micrographs showing immunogold labelling for mitochondrial proteins in ultrathin sections of club cells in newborn (NB), P15, and adult (AL) animals. Lung tissue processed for immunoelectron microscopy was incubated with gold-labelled secondary antibody particles and thereafter contrasted with uranyl acetate and lead citrate prior to analysis by transmission electron microscopy. (a–i) Immunogold labelling in mitochondria of club cells for (a–c) complex III (UQCR2), (d, e) complex IV (COX1), and (g, h) complex V (ATP6E). *S*, secretory granule. Bars: a, c, e, g, and i = 0.5 *μ*m and b, d, f, and h = 0.25 *μ*m.

To characterize the differential abundance of mitochondrial encoded proteins, immunoblot analysis of distinct mitochondrial proteins in lung homogenates from newborn, P15, and adult animals was performed. The results revealed a significant and continuous increase in the abundance of polymerase gamma 2 (POLG2), ATP synthase (ATP5b), and cytochrome oxidase subunit II (COX2) from the newborn to adult lungs with high levels of abundance in adult lungs in comparison to the low levels of abundance in the lungs of newborn animals (Figures 1(a) and 1(b)). Similarly, cytochrome oxidase subunit I (COX1) also showed a continuous upregulation as was observed in P15 and adult lungs in comparison to the newborn lungs. However, the protein abundance of COX1 detected in the newborn was higher than the ones for COX2 as well as ATP synthase and POLG2. Interestingly, the expression of NADP dehydrogenase complex I subunit I (MT-ND1) was only present in very low amounts in the lungs of newborns but increased thereafter to a still relatively low level in adults. The succinate dehydrogenase complex II subunit D (SDHD) was differently altered. It exhibited a higher abundance level in the newborn in comparison to all other mitochondrial proteins. Similar to other mitochondrial proteins, it was upregulated at P15 lungs but decreased thereafter to an intermediate level in adult animals (Figures 1(a) and 1(b)). The reason for not presenting blots for complex III is that the antibody did not work in Western blots, whereas it worked perfectly well in morphology for immunogold labelling (Figures 2(d)–2(f)), suggesting that this antibody mainly detected the native (nondenatured) protein.

### 3.2. Relative mRNA Expression of Different Complex I Gene Subunits during Postnatal (Newborn, P15, and Adult) Development of the Mouse Lung

**Figure 3:**
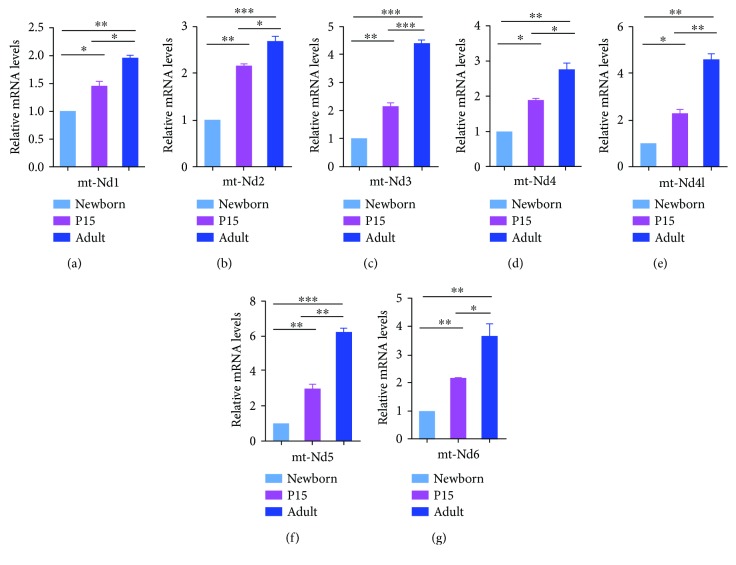
mRNA expression of the mitochondrially encoded complex I genes in the new born, P15, and adult lung tissues as determined via quantitative real-time PCR (qRT-PCR) analysis. Total RNA was extracted from the lung tissues of newborn (NB), P15, and adult (AL) mice using RNAzol, reverse transcribed and subjected to qRT-PCR using specific primers for the mRNAs for distinct subunits of the mitochondrially encoded complex I (*mt-Nd1*, *mt-Nd2*, *mt-Nd3*, *mt-Nd4*, *mt-Nd4l*, *mt-Nd5*, and *mt-Nd6*). The bar graphs represent relative levels of the transcripts in three independent experiments. *p* values were calculated by the one-way ANOVA using Tukey's test. Statistical significance is indicated by ^∗^ *p* < 0.05, ^∗∗^ *p* < 0.01, and ^∗∗∗^ *p* < 0.001. The mRNA levels were normalized to the ones of GAPDH.

To ascertain the expression of genes encoding mitochondrial proteins during postnatal development, total RNA was isolated from 3 lung tissue samples of newborn, P15, and adult mice and subsequently analysed by quantitative polymerase chain reaction (qRT-PCR). These results revealed a continuous and significant increase in the expression of all complex I genes (*mt-Nd1*, *mt-Nd2*, *mt-Nd3*, *mt-Nd4*, *mt-Nd4l*, *mtNd5*, and *mt-Nd6*) (Figures 3(a)–3(g)). This mRNA upregulation was observed in the lungs from both P15 and adult animals in comparison to the lungs from the newborn mice. Similarly, the mRNA expression of these genes showed a significant elevation in the lungs from adult animals in comparison to the lungs from the P15 mice. Interestingly, the *mt-Nd5* (Figure 3(f)) showed the highest expression among the other complex I subunits, where an increase of 2.5 and 6 times was observed in the lungs from P15 and adult animals, respectively, as compared to the neonates. The second highest increase in the gene expression was detected for *mt-Nd3* (Figure 3(c)), *mt-Nd4l* (Figure 3(e)), and *mt-Nd6* (Figure 3(g)) genes where a 2 and 4 times increase in the mRNA levels was observed in the lungs of the 15-day and 12-week animals, respectively. The real-time PCR results showed that *mt-Nd2* and *mt-Nd4* (Figures 3(b) and 3(d)) genes were much less upregulated (by approximately 2 and 2.8 times) in P15 and adult lungs, respectively, in comparison to *mt-Nd3*, *mt-Nd4l*, and *mtNd5* (Figures 3(c), 3(e), and 3(f)). The expression of complex I subunit 1 gene *mt-Nd1* (Figure 3(a)) was the lowest among the other complex I subunits by only showing an increase of by 1.5 and 2 times in the P15 and adult animals, respectively, in comparison to the newborns.

### 3.3. Relative Gene Expression Levels for Complex II–V mRNAs during Different Stages of the Postnatal Mouse Lung Development

**Figure 4:**
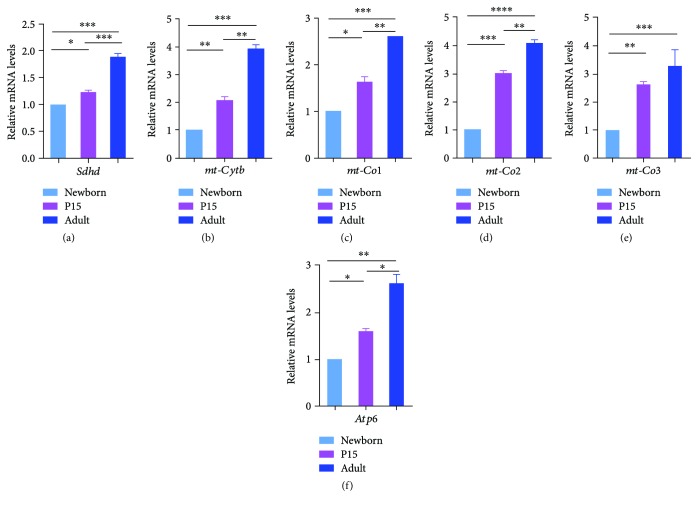
mRNA expression of the complex II–V genes in new born, P15, and adult lung tissues as determined by real-time PCR analyses. Total RNA was extracted from lung tissue of newborn (NB), P15, and adult (AL) mice using RNAzol, reverse transcribed and subjected to qRT-PCR using specific primers for mRNAs of (a) the nuclear-encoded succinate dehydrogenase complex II subunit D (*Sdhd*), (b) the mitochondrially encoded complex III (*mt-Cytb*), (c–e) the different complex IV subunits (*mt-Co1*, *mt-Co2*, and *mt-Co3*), and (f) the mitochondrially encoded complex V (*mt-Atp6*). The bar graphs represent relative levels of the transcripts in three independent experiments. *p* values were calculated by the one-way ANOVA using Tukey's test. Statistical significance is indicated by ^∗^ *p* < 0.05, ^∗∗^ *p* < 0.01, ^∗∗∗^ *p* < 0.001, and ^∗∗∗∗^ *p* < 0.0001. The mRNA levels were normalized to the ones of GAPDH.

Real-time PCR results of the total lung RNA content revealed a continuous increase in the expression of the mRNA for the nuclear-encoded succinate dehydrogenase subunit D (*Sdhd*) (Figure 4(a)), mitochondrially encoded cytochrome b (*mt-Cytb*) (Figure 4(b)), mitochondrially encoded cytochrome c oxidase subunits I and II (*mt-Co1* and *mt-Co2*) (Figures 4(c) and 4(d)), and mitochondrially encoded ATP synthase 6 (*mt-Atp6*) (Figure 4(f)) from newborn to adult stage. Complex II *Sdhd* (Figure 4(a)) exhibited the lowest but still significant increase of gene expression among all mRNAs for respiratory complexes. The mRNA expression level for complex III (*mt-Cytb*) (Figure 4(b)) was strongly upregulated (4 times) in the lungs of adults in comparison to the lungs of the newborns. Interestingly, the expression of the mRNAs for the three mitochondrially encoded complex IV subunits were altered differently. The *mt-Co2* (Figure 4(d)) was the most highly expressed among the other 2 subunits since it already increased 3 times in the lungs from P15 animals and up to 4 times in the adult lungs in comparison to the situation in the lung samples from the newborns. The mRNA level for *mt-Co1* (Figure 4(c)) was only increased by 1.5 and 2.5 times in P15 and adult animals, respectively, and these expression levels were the lowest compared to the ones for complex III subunits II (*mt-Co2*) and III (*mt-Co3*). When compared to the mRNAs for respiratory complex III subunits, the mRNA level of *mt-Co3* is not induced significantly between the values of P15 and adult animals. The mRNA levels for *mt-Atp6* (Figure 4(f)) exhibited a 1.5 and 2.5 times elevation in the lungs from P15 and adult mice in comparison to newborn animals.

### 3.4. Expression of mtDNA Transcription Machinery, Mitochondrially Encoded RNAs, and Mitochondrial DNA Polymerase Genes

**Figure 5:**
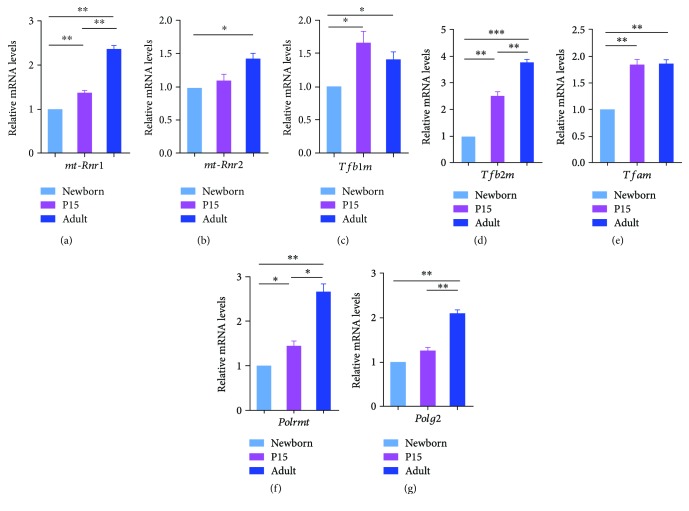
mRNA expression of the mtDNA transcription machinery genes, mitochondrially encoded RNA, and mitochondrial DNA polymerase in the new born, P15, and adult lung tissues as determined via quantitative real-time (qRT-PCR) analysis. Total RNA was extracted from the lung tissues of newborn (NB), P15, and adult (AL) mice using RNAzol, reverse transcribed and subjected to qRT-PCR using specific primers of the mtDNA transcription machinery (*Polrmt*, *Tfam*, *Tfb1m*, and *Tfb2m*), mitochondrially encoded RNAs *(mt-Rnr1* and *mt-Rnr2*), and mitochondrial DNA polymerase (*Polg2*) involved in mitochondrial DNA replication. The bar graphs represent relative levels of the transcripts in three independent experiments. Statistical significance is indicated by ^∗^ *p* < 0.05, ^∗∗^ *p* < 0.01, and ^∗∗∗^ *p* < 0.001. The mRNA levels were normalized to the ones of GAPDH.

Real-time PCR results revealed that the rRNA expression patterns of genes encoding mitochondrial RNA were distinct from each other. A 1.4 and 2.4 times increase in the *mt-Rnr1* (Figure 5(a)) expression was observed in the lungs from P15 and adult mice, respectively. The second mitochondrial encoded rRNA, *mt-Rnr2* (Figure 5(b)), only showed a 1.4 times increase in the lungs of adult animals in comparison to the lungs of newborn mice whereas no significant change in the rRNA expression of this gene was observed in the P15 lungs in comparison to the newborn and adult animals. *Tfb1m* and *Tfam* (Figures 5(c) and 5(e)), the genes encoding transcription factors implicated in the mitochondrial DNA (mtDNA) transcription machinery, were regulated in a similar pattern with 1.7 times higher expression levels in the lungs of P15 and adult animals when compared to the newborn mice. No significant changes in the expression levels of these genes were observed between the P15 and adult stages. However, the mRNAs for *Tfb2m* and *Polrmt* (Figures 5(d) and 5(f)), which encode proteins involved in the mtDNA transcription machinery, showed a significant continuous postnatal elevation in the mRNA expression. The expression of mitochondrial DNA polymerase *Polg2* (Figure 5(g)) was differentially altered, exhibiting only a significant 2-time elevation in the mRNA levels in the adult lungs in comparison to the ones of newborn and P15 stages.

### 3.5. Imaging of Mitochondrial Structural Alterations by Transmission Electron Microscopy (TEM)

**Figure 6:**
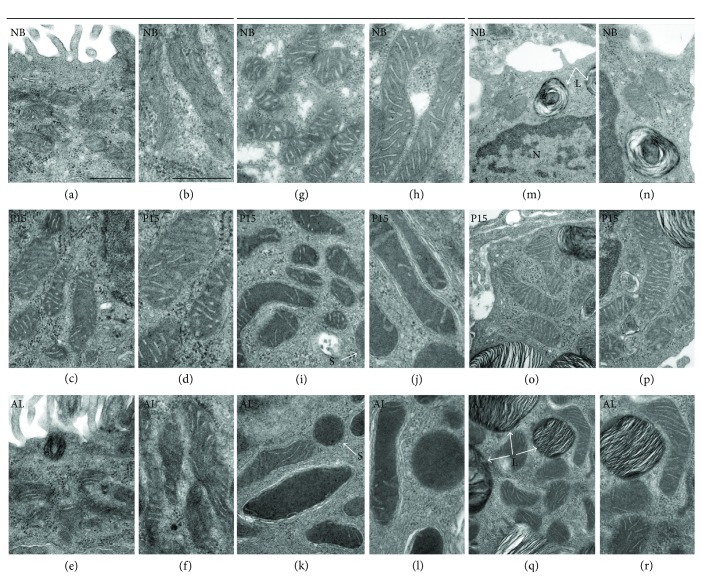
Transmission electron micrographs of mitochondrial ultrastructure in the ciliated cells, club cells, and alveolar epithelial type II cells (AECII) from the lungs of newborn (NB), P15, and adult (AL) animals. Ultrathin sections of lung samples for routine electron microscopy were contrasted with uranyl acetate and lead citrate prior to analysis by transmission electron microscopy. The left panel (a–f) represents the TEM images of ciliated cells, the middle panel (g–l) represents the club cells, and the right panel (m–r) represents the AECII cells. Higher magnifications of selected areas (b, d, f, h, j, l, n, p, and r). L, lamellar body; N, nucleus; S, secretory granule. Bars: a, c, e, g, i, k, m, o, and q = 0.5 *μ*m and b, d, f, h, j, l, n, p, and r = 0.25 *μ*m.

To visualize the mitochondrial ultrastructure in the uranyl acetate and lead citrate, contrasted ultrathin lung tissue sections from newborn, P15, and adult mice transmission electron microscopy (TEM) were applied. The lung tissue from the newborn (NB), P15, and adult (AL) animals exhibited normal ultrastructure revealing alterations in the organization of mitochondria in the ciliated cells (left panel), club cells (middle panel), and alveolar epithelial type II (AECII) cells (right panel) in different stages of postnatal development. The micrographs of the ciliated cells (left panel) from the newborns (Figures 6(a) and 6(b)) show many mitochondria with lamellar cristae in the apical part of the cell directly underneath of the basal bodies to which cilia are attached. Additionally, the pools of glycogen were observed in the cytoplasm of the ciliated cells from the newborns. Starting from P15, the mitochondria changed to larger and elongated structures with more prominent cristae and a decline in the glycogen content was well observed postnatally.

Figure 6 (middle panel) reveals the typical organelle distribution in the club cells. The micrographs of the club cells from the newborns (Figures 6(g) and 6(h)) revealed elongated mitochondria possessing lamellar cristae. Besides the mitochondria, a substantial part of the cytoplasm in club cells of newborn animals was filled with glycogen deposits. Almost no evidence of secretory activity was detected in the club cells of the newborns, whereas secretory granules were present in P15 (Figures 6(i) and 6(j)) animals. These data show distinct cell-autonomous ultrastructural changes in the club cells of newborn (Figures 6(g) and 6(h)) and P15 (Figures 6(i) and 6(j)) animals. The mitochondria in these cells were more numerous but contained fewer cristae. Moreover, the cytoplasmic glycogen—although still present in high amounts—appeared to be reduced. Electron-dense secretory granules (S) were clearly observed in the club cells of P15 animals suggesting secretory activity. The micrographs of the club cells in adult animals (Figures 6(k) and 6(l)) revealed large and elongated mitochondria in the club-formed apex which were almost devoid of cristae. Moreover, these micrographs demonstrated an increase in the abundance of secretory granules and a strong decrease in the proportion of cytoplasmic glycogen in the adult club cells in comparison to the 2 previously mentioned postnatal stages.

The micrographs of the right panel depict typical ultrastructure of the alveolar epithelial type II cells (AECII). Similar to the ultrastructure of club cells, AECII of the newborn animals (Figures 6(m) and 6(n)) showed an abundant glycogen content in their cytoplasm. The mitochondria, during this stage of postnatal development, appeared as single, spherical structures. Moreover, only few whirl-shaped lamellar bodies were detected in the cytoplasm of AECII at this stage. Starting from P15 (Figures 6(o) and 6(p)), the mitochondria changed in shape into a more elongated and branched structures with a more complex lamellar cristae as was also seen in adult animals (Figures 6(q) and 6(r)). In addition to this, alveolar epithelial type II cells of P15 to adult animals showed a gradual decline in the cytoplasmic glycogen amount. Despite a decline in glycogen content, the lamellar body number, size, and mature appearance with parallel lamellae gradually increased up to the adult stage.

### 3.6. Immunofluorescence Staining for Complex IV Subunits I and II

**Figure 7:**
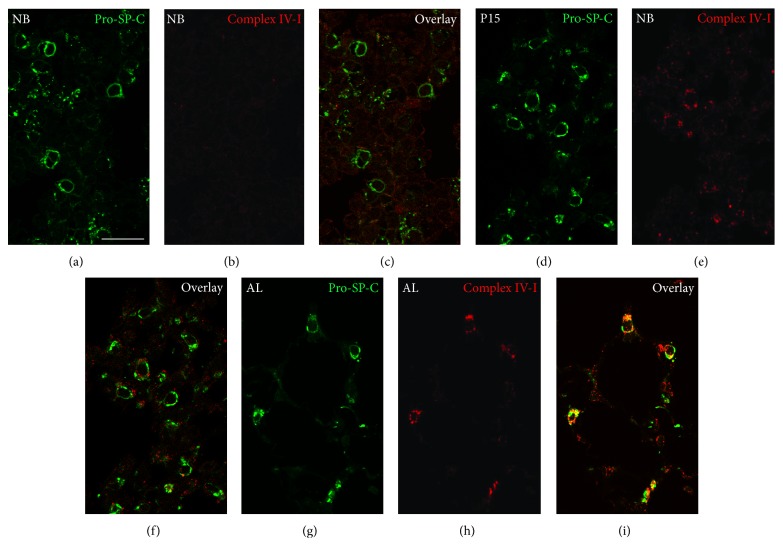
Representative immunofluorescence analysis of mitochondrial complex IV subunit I protein in AECII from lung tissue sections of newborn (NB), P15, and adult (AL) animals. Lung tissue samples from the three postnatal stages were embedded into paraffin. Thereafter, 3 *μ*m paraffin sections were cut with a rotation microtome and processed further for indirect double immunofluorescence. The lung sections were incubated overnight for double labelling with primary antibodies against mitochondrial complex IV subunit I and pro-SP-C, a marker for AECII (Table 1). The following morning, the sections were washed and incubated with the secondary antibodies (Table 1) for 2 h at room temperature. Double fluorescence samples were analyzed by confocal laser scanning microscopy (CLSM) with a Leica TCS SP5. (a, d, and g) Double IF stainings of AECII with their marker protein pro-SP-C. (b, e, and h) IF preparations for the mitochondrial complex IV subunit I. (c, f, and i) Double IF overlay for complex IV subunit I combined with pro-SP-C. NB, newborn; P15, postnatal day 15; AL, adult. Bars represent 20 *μ*m.

**Figure 8:**
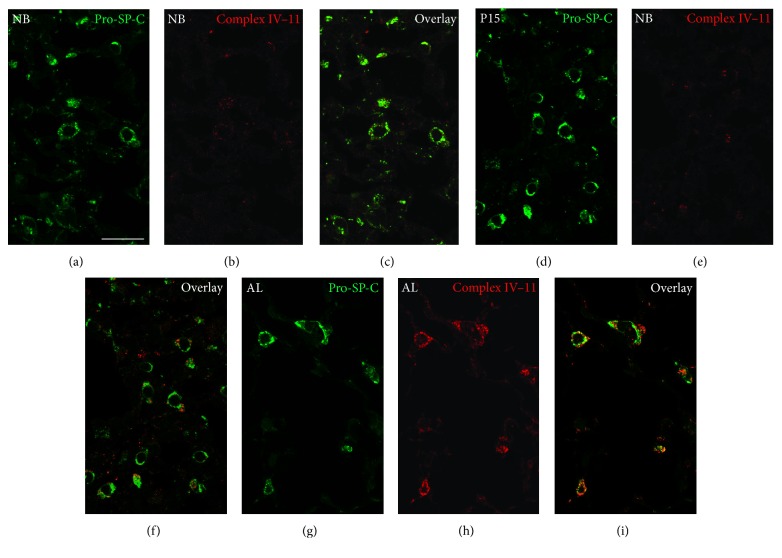
Representative immunofluorescence analysis of mitochondrial complex IV subunit II protein in AECII from lung tissue sections of newborn (NB), P15, and adult (AL) animals. Lung tissue samples from the three postnatal stages were embedded into paraffin. Thereafter, 3 *μ*m paraffin sections were cut with a rotation microtome and processed further for indirect double immunofluorescence.The lung sections were incubated overnight for double labelling with primary antibodies against mitochondrial complex IV subunit II and pro-SP-C, a marker for AECII (Table 1). The following morning, the sections were washed and incubated with the secondary antibodies (Table 1) for 2 h at room temperature. Double fluorescence samples were analyzed by confocal laser scanning microscopy (CLSM) with a Leica TCS SP5. (a, d, and g) Double immunofluorescence stainings of AECII with their marker protein pro-SP-C. (b, e, and h) IF preparations for the mitochondrial complex IV subunit II. (c, f, and i) Double IF overlay for complex IV subunit II combined with pro-SP-C. NB, newborn; P15, postnatal day 15; AL, adult. Bars represent 20 *μ*m.

Immunofluorescence preparations of lung tissue samples from newborn (NB), P15, and adult (AL) mice showed an increase in the abundance of the mitochondrial complex IV subunit I in mitochondria in AECII during postnatal development. The abundance of complex IV subunit I gradually increased exhibiting the highest levels in the type II cells of the adult lungs (Figures 7(g)–7(i)) in comparison to the lungs from P15 (Figures 7(d)–7(f)) and newborn mice (Figures 7(a)–7(c)). Despite lower abundance of mitochondrial complex IV subunit I, when compared to the mitochondrial complex IV subunit II, a gradual increase was observed in the newborn (Figures 8(a)–8(c)) and P15 animals (Figures 8(d)–8(f)). High levels of complex IV subunit II were noted in mitochondria of AECII in adult animals (Figures 8(g)–8(i)). These differences in complex IV subunit abundance as observed in the IF preparations completely corroborated the Western blot results (Figure 1(a)) in lung tissue homogenates.

### 3.7. Postembedding Immunoelectron Microscopy for Different Mitochondrial Respiratory Complexes

**Figure 9 fig9:**
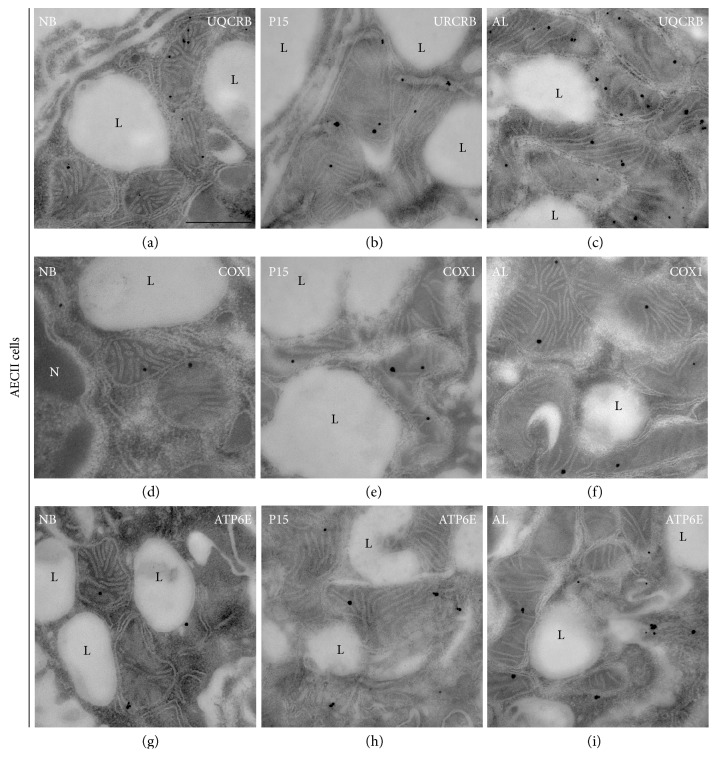
Electron micrographs showing immunogold labelling for mitochondrial proteins in ultrathin sections of AECII in newborn (NB), P15, and adult (AL) animals. Lung tissue processed for immunoelectron microscopy was incubated with gold-labelled secondary antibody particles and thereafter contrasted with uranyl acetate and lead citrate prior to analysis by transmission electron microscopy. (a–i) Immunogold labelling in mitochondria of AECII for (a–c) complex III (UQCR2), (d, e) complex IV (COX1), and (g, h) complex V (ATP6E). *S*, secretory granule; *L*, lamellar bodies. Bars represent 0.5 *μ*m.

**Figure 10:**
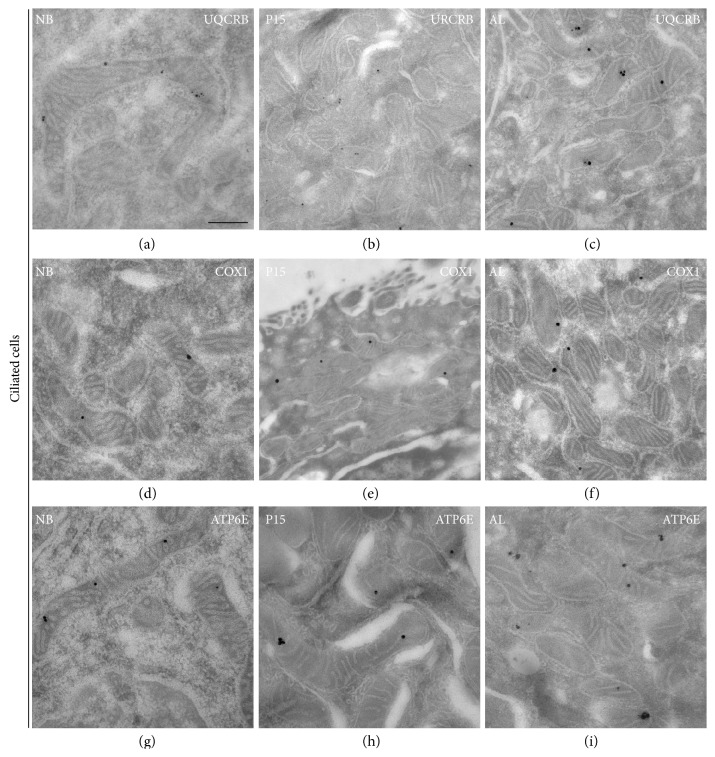
Electron micrographs showing immunogold labelling for mitochondrial proteins in ultrathin sections of ciliated cells in newborn (NB), P15, and adult (AL) animals. Lung tissue processed for immunoelectron microscopy was incubated with gold-labelled secondary antibody particles and thereafter contrasted with uranyl acetate and lead citrate prior to analysis by transmission electron microscopy. (a–i) Immunogold labelling in mitochondria of ciliated cells for (a–c) complex III (UQCR2), (d, e) complex IV (COX1), and (g, h) complex V (ATP6E). Bars represent 0.5 *μ*m.

In order to analyze the abundance of mitochondrial respiratory complex proteins on the ultrastructural level in individual mitochondria in lung tissue samples from newborn, P15, and adult mice, LR white-embedded lung ultrathin sections were processed by postembedding immunocytochemistry with the protein immunogold method and thereafter analyzed by TEM. As shown in (Figures 2, 9 and 10), gold particles clearly label mitochondria in the vicinity of their cristae, suggesting a high specificity of the antibodies used.

Due to the low abundance of many respiratory complexes in newborn animals and the use of the postembedding technique allowing mainly surface labelling, only few gold particles were present in mitochondria of AECII and ciliated cells in newborn mice (Figures 9-10(a), 10(d), and 10(j)). The gold particle number in mitochondria of club cells was even lower, resulting in labelling of few club cell mitochondria in newborn mice (Figures 2(a), 2(b), and 2(d)). A continuous gradual increase of the gold particle-labelling density occurred during later stages of postnatal development of AECII exhibiting the highest labelling density in the stainings with the antibody against complex III (UQCR2) (Figures 9(b) and 9(c)). In contrast to AECII, club cell (Figures 2(b) and 2(c)) and ciliated cell (Figures 10(b) and 10(c)) mitochondria showed significantly lower labelling for UQCR2 especially in the adult mice. We also noticed a postnatal gradual increase of the gold particle-labelling density for complex IV subunit I (COX1) in club cells (Figures 2(e) and 2(f)), AECII (Figures 9(e) and 9(f)), and ciliated cells (Figures 10(e) and 10(f)). The intensity of the labelling for complex IV in all the three cell types was the lowest in comparison to that of complexes III and V. In addition to this, the gold particle labelling for complex V (ATP6E) as well increased postnatally in all the three cell types with the intensity of the labelling being very high in AECII (Figures 9(h) and 9(i)) in comparison to club cells (Figures 2(h) and 2(i)) and ciliated cells (Figures 10(h) and 10(i)). The negative controls of Figures 2, 9 and 10 are shown in Supplementary Figure 1 where no primary antibody was used and the micrographs remained completely devoid of staining suggesting a high specificity of the secondary antibody.

## 4. Discussion

Nearly all cell types in the lung depend on the metabolic activity of the mitochondria for their energy supply that is generated via the mitochondrial respiratory chain and oxidative phosphorylation (OXPHOS) [58, 59]. Hence, mitochondrial dysfunction can contribute to the pathophysiology of various pulmonary diseases such as bronchopulmonary dysplasia [60–62], chronic obstructive pulmonary disease (COPD) [63–65], lung cancer [66, 67], cystic fibrosis [68, 69], and asthma [70, 71]. This understanding of the human normal lung functioning and the mechanisms behind lung disease comes often from studies utilizing lung samples or animal models. Nowadays, frequently, mice are employed in lung research due to the advantages that this species provides [72, 73]. Surprisingly, only few reports are available from the literature concerning mitochondrial biogenesis, mitochondrial function, and the regulation of mtDNA genes during the postnatal lung development in mice. Most of the previously published data on the postnatal development of the mitochondrial compartment were described in other organs such as the liver, heart, blood lymphocytes, brain, skeletal muscle, or cell types. Therefore, we investigated the differential expression and cell type specific differences of distinct mitochondrial respiratory chain proteins and factors of the mitochondrial transcription and translation machinery during postnatal development of the mouse lung.

### 4.1. General Ultrastructure and Alterations of the Mitochondrial Compartment during Postnatal Development of Different Cell Types in the Lung as Revealed by Transmission Electron Microscopy

Clear differences were noted in general ultrastructure and the development of the mitochondrial compartment in distinct lung cell types (ciliated, club, and AECII) of the bronchiolar or the alveolar epithelium during postnatal mouse lung development. Similar to the club and AECII cells, TEM analysis of the ciliated cells revealed an elongation and an increase in the size of mitochondria starting from P15 in comparison to the newborns. In addition, this postnatal development of the ciliated cells was accompanied by a decrease in the cytoplasmic glycogen deposits. These morphological changes could imply the postnatal development of the ciliated cells. It was reported by Francis and coworkers [74] that mice are born with few ciliated cells in the trachea and that there is a postnatal increase in the ciliated cell density and cilia-generated flow in the trachea of the C57BL/6 mice from postnatal day 0 to day 28. Another study by Sorokin [75] described that the earliest ciliary motion was observed at postnatal days 5 to 7 in culture series of fetal rat lungs. Additionally, ciliary vibration after first being detected in the trachea and largest bronchi appears in the epithelium of smaller bronchial branches coinciding with the pattern of epithelial differentiation in the fetal lung [76]. Furthermore, our micrographs showed the distribution of mitochondria directly underneath the apical part of the cell, below the cilia. This distribution of mitochondria is necessary for the mammalian ATP-dependent ciliary beating [77].

TEM analysis of club cell ultrastructure in adult animals which showed a clear decrease in the proportion of cytoplasmic glycogen and a marked increase in the abundance of secretory granules in comparison to the club cells of the newborns reflecting their higher degree of maturation in comparison to newborn club cells. This finding is in accordance with studies from Plopper et al. [30] on the rabbit lung as well as from Baskerville [78] on the pig lung, reporting a decrease in the cytoplasmic glycogen abundance and a significant increase in the amount of electron-dense secretory granules postnatally. Plopper and coworkers suggested that the glycogen degradation would be required for energy production in order for a cell to initiate the important biogenesis processes such as biosynthesis of secretory products [30]. The cellular constituents of the club cells undergo significant shifts in organelle and glycogen deposit during differentiation, suggesting a metabolic shift from glycolysis to higher respiration. In addition to these signs of cellular maturation, we detected large and dense mitochondria which became more elongated but lost their cristae in the adult mouse club cells as compared to the P15 and newborn groups which is in agreement with a study in rat lung reporting that the mitochondria from adult club cells were more devoid of cristae in comparison to the neonates [79]. Newborn and P15 animals in our study showed a comparable ultrastructure, except for the appearance of secretory granules and a decrease in the number of cristae in the mitochondria of the club cells of P15 mice. The total number and volume of the club cells, the abundance and expression of the club cell secretory protein (CC10), and the volume of lung and bronchioles increased postnatally in P15 and adult mice in comparison to the newborns suggesting that club cell maturation occurs postnatally [54]. Collectively, all these findings reveal the postnatal differentiation and maturation of the club cells in the mouse lung.

Ultrastructural analysis of AECII also revealed a gradual decrease in glycogen amounts during the postnatal development. Additionally, our micrographs showed an increase in the number of lamellar bodies from the newborns to the P15 and adult animals. These results are in accordance with other findings on the development and maturation of AECII in the rat model [29]. Moreover, our data showed an increase in the mitochondrial length where more elongated mitochondria with longer and more densely packed lamellar cristae were observed in AECII from P15 and adult mice as compared to the newborn mice. These results are in agreement with published data on mitochondrial shape and volume in AECII of the lungs of Sprague-Dawley rats [29]. In previous articles using different animal species, it was suggested that this increase in the volume of mitochondria and change in its shape throughout the postnatal stages of development could be associated with the growing metabolic demands of the cell as well as with the production of a greater area-to-volume ratio [29, 32]. Furthermore, also in other organs, significant alterations of mitochondria were observed during postnatal development. For instance, Sato and colleagues detected larger and elongated mitochondria in the brain cortex of Wistar rats from postnatal day 5 with cristae reaching the maximal complexity in 15- and 21-day-old rats [52]. They speculated that this increase in the cristae complexity is correlated with the increase of respiratory enzyme activities in the membrane of the mitochondria. This change in the mitochondrial structure and shape in AECII and club cells is associated with morphological and functional differentiation of the cells as well as it is correlated with the total lung volume enlargement during the postnatal development. Similar to the club cells, AECII differentiate and mature postnatally where it was shown in rats that there is an increase in the fraction volume composition of AECII subcellular organelles (cytoplasm, mitochondria, and lamellar bodies) in adult Sprague-Dawley rats in comparison to the newborns [29]. Another study confirming the postnatal maturation of AECII in mice has shown that from E17.5 to P5 differentiation and maturation of AECII occurs that is associated with the ability of the cells to secrete surfactant [80].

Indeed, in our study, we were able to prove that mitochondrial respiratory complexes, as well as transcription factors and mitochondrial ribosomal RNA, were strongly upregulated during postnatal development. In the following, our results on alterations of respiratory complexes are compared in details to the international literature.

### 4.2. Complex I

Complex I is known as the largest complex of the mitochondrial respiratory chain which transfers electrons from NADH to coenzyme Q and uses this free energy to pump protons from the mitochondrial matrix into its intermembrane space [81, 82]. The results of our study show a significant upregulation of mitochondrially encoded complex I gene as well as protein (MT-ND1) starting from the lungs of P15 mice and reaching the highest peak in the lungs of the adult mice. Comparable to our results, Bates and colleagues [51] detected a significant increase in the activity of complex I in the rat brain from postnatal day 1 to day 21, suggesting that the increase is due to the high demand for mitochondrial ATP during the brain development. Interestingly, not only mitochondrially encoded complex I subunits increased in the expression during the postnatal development as was shown by our study but also the nuclear-encoded mitochondrial subunits as was revealed by Wirtz and Schuelke [53]. In their study on the expression of the 33 nuclear-encoded complex I genes during postnatal development of the C57BL/6J mouse brain, they found the rise of expression intensity of the complex I around P11 in comparison to earlier stages and this coincided with the synaptogenesis [53, 83–85]. A more recent study of the mitochondria in the intrinsic muscle of Wistar rat tongue demonstrated a gradual increase in the mRNA expression of the mitochondrially encoded complex I subunit *mt-Nd1* from birth to 15 days of age reaching the highest expression at 21 days of age [44]. Comparable results were observed from Fujita and Sato [44] for the level of NADH-O_2_ oxidoreductase activity in the intrinsic muscle of the Wistar rat tongue. They hypothesized that the hyperactivity in the NADH enzyme is indispensable for the transition from swallowing to mastication and that the increase in *mt-Nd1* could be a result of this hyperactivity [44].

### 4.3. Complex II

Complex II is composed of 4 subunits which are encoded solely by nuclear DNA, with succinate dehydrogenase (SDH) being the largest subunit. This complex forms a direct link between the tricarboxylic acid (TCA) cycle and the respiratory chain [86]. Surprisingly, the only exception of the general mitochondrial maturation pattern was observed in our study for succinate dehydrogenase subunit D (SDHD) of complex II. We found that SDHD protein abundance peaked in the lung tissue samples from P15 mice and then decreased in the lungs from the adult animals. Moreover, although the mRNA expression of *Sdhd* was found to gradually increase starting from the lungs of P15 animals, the relative increase of this expression was the lowest among all the 5 complexes studied. Interestingly, our data are in agreement with the study by Valcarce and colleagues [28] that showed a strong increase in the postnatal SDH activity in the liver of albino Wistar rats until postnatal hour 6, and afterwards, the activity of the SDH started to decline. It was suggested that this increase in mitochondrial enzyme activity is due to the increased rate of protein synthesis for mitochondrial enzymes after birth [28]. In a more recent study, Sato and colleagues [52] demonstrated as well an upregulation in the activity of SDH, from the Wistar rat brain cortex that increased at 15 days postnatal, maintained at 21 days, and then decreased in mitochondria of adult animals. The reason behind this is not clearly understood, but several other studies showed a similar pattern of SDH activity. For instance, Sieck and Blanco [87] reported in their study on the postnatal changes in the succinate dehydrogenase activity in the diaphragm of cats that the muscle fiber SDH activity increased between 3 and 6 weeks postnatally declining thereafter to adult values. In addition, in a study on the postnatal development of complex II in mitochondria isolated from rat brain synaptosomes, an increase in the activities of complex II from day 10 to day 15, without significant alterations of SHD activity thereafter, was reported [88].

### 4.4. Complex III

Complex III constitutes the central part of the mitochondrial respiratory chain and is composed of 11 different subunits where the cytochrome b gene *mt-Cyb* is the only complex III subunit encoded in the mitochondrial DNA (mtDNA) [89, 90]. Our data revealed a significant gradual upregulation in the expression of the *mt-Cytb* mRNA in the lungs of the mice starting from postnatal day 15 (P15) in comparison to the lungs from the newborn animals. Our findings are in agreement with the results of the study from Marin-Garcia and colleagues [49] where they detected a 3- and 4-fold increase in the mRNA expression of *mt-Cytb* in the bovine hearts of late fetal and young adult stages, respectively, in comparison to early fetal stages of development which correlated with the increase in mtDNA copy number. In addition to this, Schagger et al., in 1995, showed that the total protein amount of complex III and its catalytic activity was increased in the heart and liver tissue samples of Wistar rats during transition from fetal to adult stage correlating with the higher demands of oxidative phosphorylation activities in proliferating tissues [47]. Complex III activity was as well significantly upregulated in the mitochondria from the rat brain from postnatal day 1 to day 60 as was reported by Bates and colleagues [51].

### 4.5. Complex IV

Complex IV transfers the electrons from cytochrome c to molecular oxygen driving downstream ATP biosynthesis. It consists of 14 subunits, three of which (*mt-Co1*, *mt-Co2*, and *mt-Co3*) are encoded by mitochondrial genome [91]. In our study, we analyzed the three mitochondrially encoded subunits (subunits I–III) of complex IV at the mRNA (*mt-Co1*, *mt-Co2*, and *mt-Co3*) and protein (COX1, COX2) level and we found that the mRNA expression and abundance of this complex is drastically increased starting from the lungs of P15 mice. However, the increase in the abundance of these subunits, as shown by immunofluorescence and Western blot results, exhibited a different pattern of upregulation. The abundance of complex IV subunit I gradually increased displaying the highest levels in the type II cells of the adult lungs in comparison to the lungs from P15 and newborn mice. Similarly, a gradual increase in mitochondrial complex IV subunit II was as well observed; however, newborn and P15 animals exhibited lower levels of this protein in comparison to the labelling for subunit I. The increase in the expression and abundance of complex IV was the highest in comparison to other mitochondrial respiratory complexes. Moreover, we detected that complex IV, although upregulated in club and AECII cells, showed different levels of upregulation between these lung cell types as was shown by electron microscopy. There was a continuous gradual increase of the gold particle labelling during later stages of postnatal development; however, in contrast to AECII, club cell mitochondria exhibited significantly lower labelling for complex IV especially in adult mice. Our results are in accordance with other studies which as well detected the elevation of the complex IV during postnatal development of distinct species such as in the (1) Wistar rat hearts by Schagger and colleagues [47], (2) rat brain by Almeida et al. [88], (3) Wistar rat heart homogenates by Drahota and colleagues [48], (4) bovine heart tissues by Marin-Garcia and colleagues [49], and (5) liver and muscle COX activity in human samples by Pejznochova and colleagues suggesting that the maturation of the mitochondrial compartment is important for the differentiation of tissues and organs in mammals [92].

### 4.6. Complex V

Mitochondrial complex V, ATP synthase, is responsible for the synthesis of ATP from the ADP in the mitochondrial matrix using the provided energy from the proton electrochemical gradient [93, 94]. Nearly no labelling for complex V was detected in the club cells of the newborn mice in comparison to the AECII in the newborns. Moreover, complex V showed a lower labelling in the club cell mitochondria of the adult mice as compared to the labelling in the AECII of adult animals. Our data on complex V are in agreement with a study in which isolated brain mitochondria of rats showed a significant increase in the activity of complex V starting from postnatal day 10 in comparison to the postnatal day 1 [51]. Similarly, Marin-Garcia and colleagues showed an increase in the expression of *ATP-β Synthase* mRNA in the bovine heart tissue from the late fetal and young adult animals [49].

### 4.7. Associated Mitochondrial Transcription Machinery and Mitochondrial rRNAs

It is known that the initiation of mitochondrial transcription needs nucleus-encoded proteins, such as POLRMT, auxiliary factors for promoter recognition, such as TFB1M and TFB2M, and promoter activation like Tfam [95–97]. These factors are required for the transcription of mtDNA that controls the rRNA/mRNA ratio. We have noticed that the regulation of the machinery required for the maintenance and expression of mtDNA showed a significant increase from postnatal day 15 to the adult stage in comparison to the newborn mice. These results are in accordance with earlier study showing a postnatal increase in the mtDNA content in the mouse lung during the first 2 months, followed by a moderate decline at 5 months and then an increase again by 15 months of age [98]. This increase in mtDNA level is associated with periods of increased susceptibility of the tissues to oxidative stress and injury during the early life and advance stages [98]. Likewise, an increase in the expression of the mitochondrial transcription factor (TFB2M) and the mitochondrial RNA polymerase (POLRMT) in growing rat hearts was shown in adult and aged animals [99] suggesting of an increase is the mitochondrial biogenesis in maturing cardiomyocytes which fits the role of TFB2M as an accessory subunit of POLRMT required for promoter recognition [100, 101]. However, in contrast to our study, they showed that RNA expression and protein abundance of TFAM remained constant throughout life and this might be due to the fact that TFAM levels are correlated with the amount of mtDNA in the cell [99]. TFAM is an mtDNA-binding protein required for the transcription of mtDNA and is thought to act as mtDNA copy number regulator [102, 103]. It was suggested, in this study, that rats, apparently, do not need an increase in mtDNA in order to cope with increased mitochondrial biogenesis during the postnatal heart development. In addition to this, Pohjoismäki and colleagues [99] also showed an elevation in the expression of *Polg2* in 10-day-old rats as compared to neonates. Mitochondrially encoded RNA (rRNA) is necessary for mitochondrial protein biosynthesis. After mtDNA is transcribed, mitochondria translate their mRNA on mitochondrial ribosomes consisting of 2 mtDNA-encoded rRNAs and nuclear-encoded proteins [104]. Our results report an increase in the expression of mitochondrial rRNA during the postnatal development of murine lungs. This increase in the expression is reasonable as it emphasizes the upregulation of the mitochondrial translation machinery and mitochondrial expression systems during the postnatal development showing that the cells are still proliferating.

## 5. Conclusions

In this article, we show that the alterations of the mitochondrial compartment with special focus on mitochondrial respiratory complexes and associated mitochondrial transcription machinery as well as mitochondrial rRNAs exhibited a specific adaptation and differential maturation of the mitochondrial compartment according to the metabolic needs of individual cell types during postnatal development of the mouse lung.

## Figures and Tables

**Table 1:** List of primary and secondary antibodies used in this study.

Primary antibody against antigen	Symbol	Host	Company	Cat. number	Dilution (WB)	Dilution (IF)	Dilution (IEM)
*Mitochondrial proteins*							
NADH:ubiquinone oxidoreductase core subunit 1 (complex I)	MT-ND1	Rabbit, polyclonal	Abcam	ab74257	1 : 500	—	—
Succinate dehydrogenase complex subunit D (complex II)	SDHD, CybS	Rabbit, polyclonal	Millipore	ABT110	1 : 3000	—	1 : 50
OXPHOS complex III core 2 subunit (complex III)	UQCR2	Mouse, monoclonal	Invitrogen	A11143	—	1 : 250	—
OXPHOS complex IV subunit I/cytochrome c oxidase I (complex IV)	COX1, MT-CO1	Mouse, monoclonal	Invitrogen	459600	1 : 1000	—	1 : 100
OXPHOS complex IV subunit II/cytochrome c oxidase II (complex IV)	COX2, MT-CO2	Mouse, monoclonal	Invitrogen	A6404	1 : 1000	—	—
ATP synthase, H^+^ transporting mitochondrial F1 complex, beta subunit (complex V)	ATP5b	Mouse, monoclonal	Life Technologies	A21351	1 : 3000	—	—
ATP synthase 6 (complex V)	MT-ATP6E	Rabbit, polyclonal	Santa Cruz	Sc-20946	—	—	1 : 50
Polymerase (DNA) gamma 2, accessory subunit	POLG2	Mouse, polyclonal	Abcam	ab66961	1 : 1000	—	—
Mitochondrial transcription factor A	TFAM	Goat, polyclonal	Santa Cruz	Sc-19050	—	—	—
*Cell-specific markers*							
Prosurfactant protein C	Pro-SP-C	Rabbit, polyclonal	Chemicon	ab3786	—	1 : 1000	—
Club cell protein 10 (CC10)	CC10	Rabbit, polyclonal	Santa Cruz	sc-25555	—	1 : 1000	—
*Loading control*							
Glyceraldehyde 3-phosphate dehydrogenase	GAPDH	Mouse, monoclonal	HyTest, Finland	5G4	1 : 8000	—	—
*Secondary antibodies*							
Fab anti-rabbit IgG ultrasmall gold	—	Goat	Aurion	800.255	—	—	1 : 400
Fab anti-mouse IgG ultrasmall gold		Goat	Aurion	800.266	—	—	1 : 400
Anti-rabbit-IgG Alexa Fluor 488	—	Donkey	Life Technologies	A21206	—	1 : 1000	—
Anti-mouse-IgG Alexa Fluor 555	—	Donkey	Life Technologies	A31570	—	1 : 1000	—
Anti-rabbit-IgG alkaline phosphatase conjugate	—	Goat, polyclonal	Sigma-Aldrich	A0545	1 : 20,000	—	—
Anti-mouse-IgG alkaline phosphatase conjugate	—	Goat, polyclonal	Sigma-Aldrich	A3562	1 : 20,000	—	—
*Counterstaining of nuclei for immunofluorescence*							
Hoechst 33342 (1 *μ*g/ml)	—	—	Life Technologies	33342	—	1 : 1000	—
TOTO®-3 iodide	—	—	Life Technologies	T-3604	—	1 : 1000	—

**Table 2:** List of primers used in this study for qRT-PCR (the annealing temp was 60°C).

Full name	Gene target	Sense primer (5′–3′) 20–23 mers	Antisense primer (5′–3′) 20–24 mers	Size of product
Mitochondrially encoded NADH:ubiquinone oxidoreductase core subunit 1	*mt-Nd1*	GCTTTACGAGCCGTAGCCCA	GGGTCAGGCTGGCAGAAGTAA	147
Mitochondrially encoded NADH:ubiquinone oxidoreductase core subunit 2	*mt-Nd2*	CCTCCTGGCCATCGTACTCA	GAATGGGGCGAGGCCTAGTT	124
Mitochondrially encoded NADH:ubiquinone oxidoreductase core subunit 3	*mt-Nd3*	TAGTTGCATTCTGACTCCCCCA	GAGAATGGTAGACGTGCAGAGC	100
Mitochondrially encoded NADH:ubiquinone oxidoreductase core subunit 4	*mt-Nd4*	CGCCTACTCCTCAGTTAGCCA	TGATGTGAGGCCATGTGCGA	112
Mitochondrially encoded NADH:ubiquinone oxidoreductase core subunit 4L	*mt-Nd4l*	AGCTCCATACCAATCCCCATCAC	GGACGTAATCTGTTCCGTACGTGT	109
Mitochondrially encoded NADH:ubiquinone oxidoreductase core subunit 5	*mt-Nd5*	GGCCCTACACCAGTTTCAGC	AGGGCTCCGAGGCAAAGTAT	134
Mitochondrially encoded NADH:ubiquinone oxidoreductase core subunit 6	*mt-Nd6*	CTTGATGGTTTGGGAGATTGG	ACCCGCAAACAAAGATCACC	138
Succinate dehydrogenase complex subunit D	*Sdhd*	GCTCGAGCTCTCCTACTCC	GCTTGGTGACAGGTGAATGT	117
Mitochondrially encoded cytochrome b	*mt-Cytb*	TCCTTCATGTCGGACGAGGC	AATGCTGTGGCTATGACTGCG	100
Mitochondrially encoded cytochrome c oxidase I	*mt-Co1*, *Cox1*	TCAACATGAAACCCCCAGCCA	GCGGCTAGCACTGGTAGTGA	100
Mitochondrially encoded cytochrome c oxidase II	*mt-Co2*, *Cox2*	ACCTGGTGAACTACGACTGCT	TCCTAGGGAGGGGACTGCTC	121
Mitochondrially encoded cytochrome c oxidase III	*mt-Co3*, *Cox3*	CCAAGGCCACCACACTCCTA	GGTCAGCAGCCTCCTAGATCA	150
Mitochondrially encoded ATP synthase 6	*mt-Atp6*	AGCTCACTTGCCCACTTCCT	AAGCCGGACTGCTAATGCCA	114
Mitochondrially encoded 12S RNA	*mt-Rnr1*	ACACCTTGCCTAGCCACACC	GTGGCTGGCACGAAATTTACCA	112
Mitochondrially encoded 16S RNA	*mt-Rnr2*	ACACCGGAATGCCTAAAGGA	ATACCGCGGCCGTTAAACTT	148
Transcription factor B1, mitochondrial	*Tfb1m*	GGCTGAGAGACTTGTAGCCACT	AGGTGCACCACTCCTACATCAA	150
Transcription factor B2, mitochondrial	*Tfb2m*	TTTGGCAAGTGGCCTGTGAC	ACTGATTCCCCGTGCTTTGACT	109
Mitochondrial transcription factor A	*Tfam*	GCCCGGCAGAGACGGTTAAA	GCCGAATCATCCTTTGCCTCC	137
Polymerase (RNA) mitochondrial	*Polrmt*	ACAACACCGTGATGCTTGGC	GAACATCCTGGTCCCTGCGT	150
Polymerase (DNA) gamma 2, accessory subunit	*Polg2*	CTGGTTGCGTCATCGGCTTC	TGCTTCCCTTGCGTCCCAAT	101
Glyceraldehyde 3-phosphate dehydrogenase	GAPDH	TGGCAAAGTGGAGATTGTTGCC	AAGATGGTGATGGGCTTCCCG	156

## Supplementary Materials

Supplementary Materials Supplementary Figure 1: Electron micrograph of the negative control (NC) for Figures 8 [Fig fig9]–10. (A–C) Ciliated cells, (D–F) club cells, and (G–I) AECII of newborn (NB), P15, and adult (AL) animals.
