# Supplementary figures and images for: Differential Alterations of the Mitochondrial Morphology and Respiratory Chain Complexes during Postnatal Development of the Mouse Lung
Source: Oxid Med Cell Longev. 2017 Dec 19;2017:9169146. doi: 10.1155/2017/9169146 (PMC5753018; doi:10.1155/2017/9169146)

Supplementary Figure

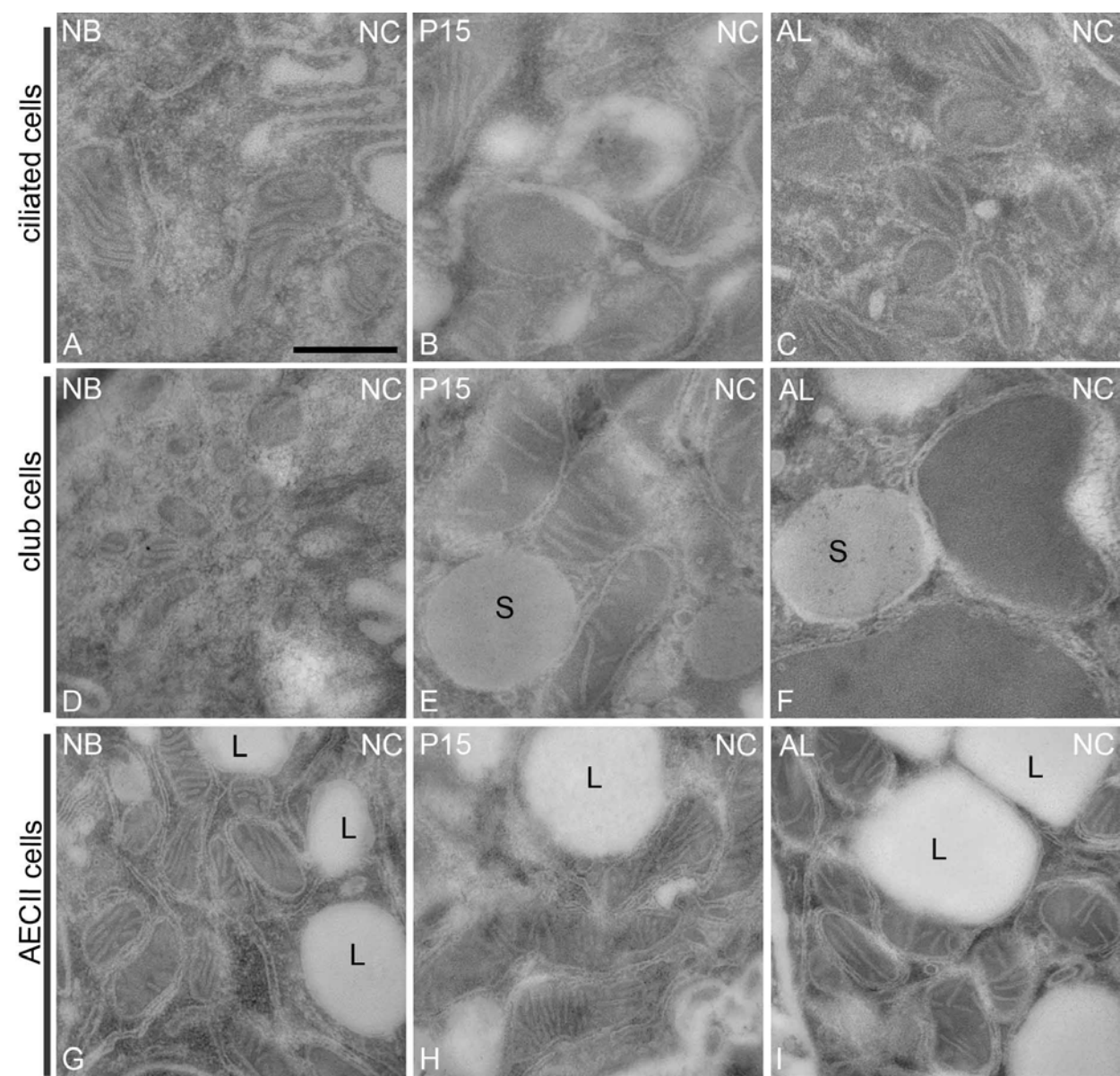

Supplementary Fig. 1.

Supplement: Supplementary Materials — Supplementary Figure 1: Electron micrograph of the negative control (NC) for Figures 8 –10. (A–C) Ciliated cells, (D–F) club cells, and (G–I) AECII of newborn (NB), P15, and adult (AL) animals. [file 9169146.f1.pdf]
